# The Effects of Anisometropic Amblyopia on the FNS and TNO Stereotest Thresholds in Four- to Eight-Year-Olds

**DOI:** 10.22599/bioj.123

**Published:** 2019-04-25

**Authors:** Aishat Ateiza, Helen Davis

**Affiliations:** 1 Gloucestershire Hospitals NHS Foundation Trust, GB; 2 University of Sheffield, GB

**Keywords:** Anisometropia, Amblyopia, Stereoacuity, Binocular, Paediatric

## Abstract

**Purpose:**

To investigate the relationship between stereoacuity and factors associated with anisometropic amblyopia in children aged 4–8 years.

**Methods:**

44 participants had their stereoacuity thresholds measured using the Frisby Near Stereotest (FNS) and the TNO Randot Stereotest (TNO). Participants were divided into anisometropic amblyopes and controls (normal uniocular visual acuity (VA) with or without glasses). FNS and TNO stereoacuity thresholds were compared based on different factors, which included interocular acuity difference (IAD), VA levels, and the degree of anisometropia.

**Results:**

All 44 participants achieved better stereoacuity with the FNS compared to the TNO (*p* = 0.045). The control group performed significantly better on the FNS (*p* = 0.012) and the TNO (*p* = 0.009) when compared with anisometropic amblyopes. The only statistically significant correlation was found between stereoacuity – as measured with FNS – and IAD (*p* = 0.009). However, the TNO showed a correlation in the presence of poor VA, larger IADs and a high degree of anisometropia.

**Conclusions:**

Stereoacuity thresholds are significantly affected by poor VA, large IAD and high degrees of spherical anisometropia when trying to distinguish the resolution of a target with the TNO yet the same factors do not appear to affect ability to distinguish the disparity of a target with the FNS. Controls also performed worse on the TNO.

## Introduction

Amblyopia has been defined as “a unilateral or bilateral decrease of vision which persists after correction of the refractive error and removal of any pathological obstacles to vision” (Ansons & Davis, 2014: 285), and has been estimated to affect 1–4% of children (Kvarnström, Jakobsson, Lennerstrand, 2002; Eibschitz-Tsimhoni et al., 2000).

Anisometropia is one of the main causes of amblyopia, characterised by a difference in interocular refractive error (Awadein & Fakhry, 2011; Sapkota, 2014; Bhatia & Pratap, 1976; Brooks, Johnson & Fischer, 1996). It is possible that children with anisometropia have it at birth or soon after (Hardman-Lea, Loades & Rubinstein, 1989), although there is no evidence to verify this. However, Donahue (2005) proposed that by 3 years of age, anisometropic amblyopia will have occurred in those who are predisposed to develop it.

Coarse stereopsis, produced by large retinal disparities can be appreciated in the presence of amblyopia, and matures by around 4 years of age. Yet fine stereoacuity continues to mature after four years of age (Leske, Birch & Holmes, 2006; Giaschi et al., 2013a). If this is the case and anisometropic amblyopia is present before the age of 3 (Donahue, 2005), we would still expect coarse stereopsis to be affected to some degree in the presence of anisometropic amblyopia.

It is well documented that a deficit in stereoacuity correlates with the level of visual acuity (VA) and the severity of anisometropic amblyopia present (Wallace et al., 2011; Weakley, 2001; Greenwood et al., 2012; Tomaç & Birdal, 2001). Dadeya, Kamlesh & Shibal (2001) induced different degrees of uniocular myopia, hypermetropia and astigmatism in 30 normal adults. Their results showed as little as 1 dioptre (D) of blur had the ability to diminish stereoacuity levels in subjects; the higher the degree of induced blur, the greater the reduction in stereoacuity when using the Titmus Fly stereotest. These studies used only one stereotest, whereas the current study compared the response of 2 different stereotests.

It is important to note the differences between real depth stereograms (e.g. the Frisby Near Stereotest [FNS] and random dot stereograms [e.g. the TNO Randot Stereotest (TNO]). For example, the TNO measures the resolution of fine texture and simulates stereoscopic depth by the horizontal displacement of 2 images; while the FNS measures real depth, due to the thickness of each plate which produces the disparity and the horizontal component and gives rise to stereopsis. The TNO is conducted with the subject wearing red/green glasses which hinders the use of both eyes together. The subject has to try and overcome this dissociation in order to distinguish the shapes within the plates, thus the TNO is more sensitive to binocular deficiencies (Garnham & Sloper, 2006). The FNS is comprised of 3 test plates with differing distances between the front and back of each plate measuring 1 mm, 3 mm and 6 mm in thickness. The circle in the 6 mm plate is easier to distinguish due to the larger displacement between both sides of the glass plate. However, motion parallax can come into play with the FNS as a result of head movement, which can simplify the test and generate much better results. Although both stereotest types measure disparity and resolution of a target to a certain extent, each stereotest measures these factors to different degrees. It has been found that demonstrable stereoacuity can even be detected in the presence of strabismus with the FNS, whereas generally stereopsis is not demonstrable in the presence of strabismus, and some cases of anisometropic amblyopia, with random dot stereograms (RDS) (Leske, Birch & Holmes, 2006; Cooper & Feldman, 1978; O’Connor et al., 2010). Simons (1981) conducted a study on children which showed that out of 3 different stereotests (FNS, TNO and Random-Dot E [RDE]), the FNS was unable to detect some cases of binocular abnormality compared to the TNO and RDE tests. It is possible that these results are due to real depth tests, like the FNS, assessing whether stereopsis is present or absent; whereas RDS, like the TNO, are more rigorous and determine more subtle changes in stereoacuity (Leske, Birch & Holmes, 2006).

There has been ample research into the effect of amblyopia on different visual systems, including binocular functions. There is an abundance of evidence that demonstrates how anisometropic amblyopia contributes to the deficit in stereoacuity, including reports on how stereoacuity improves with VA improvement after occlusion therapy (Holopigian et al., 1986; Brooks, Johnson & Fischer, 1996; Steele et al., 2006; Lee & Isenberg, 2003). Most of these studies have been conducted using kittens, primates or adults with induced monocular blur (Hubel & Wiesel, 1963; Kiorpes et al., 1987; Horton & Stryker, 1993; Oguz & Oguz, 2000; Atilla & Erkam, 2011; Singh et al., 2015). The present research will aim to provide further evidence to help determine what factors contribute to the deficit in stereoacuity seen in anisometropic amblyopes, using a paediatric population of true anisometropic amblyopes and their controls.

### Hypothesis

Controls will achieve significantly better stereoacuity with the TNO and the FNS compared to the anisometropic amblyopes.

## Method

Ethical approval was obtained from the Northern Ireland Research Ethics Committee (REC Reference: 15/NI/0260) and permission was gained from the Mid Yorkshire Hospitals NHS Trust (MYHT). All experiments and data collection were conducted in a manner compliant with the Declaration of Helsinki and informed consent was obtained from participants and/or parents.

A quantitative, cross-sectional approach was used for this study. Fifty-two participants were recruited initially, however 8 participants were excluded due to underlying pathology or microstrabismus. Of the remaining 44 participants recruited, 20 were anisometropic amblyopes and 24 were controls. Within the anisometropic amblyopes group, 11 had previously undergone or were undergoing occlusion therapy and nine were solely treated with refractive correction. Participants were aged between 4 and 8, and recruited from the patient population seen within the MYHT Orthoptic Department.

Participants were selected based on the inclusion criteria outlined below:

Anisometropic amblyopes:-

- Children between the ages of 4 and 8 who have the capacity to understand and complete the clinical tests.
- No strabismus/squint seen on cover test and no known associated pathology.
- At least 1D difference in refractive error (spherical component).
- At least 2 LogMAR lines difference in vision between both eyes, using either Crowded Kay Pictures or Crowded Keeler tests.

Controls:-

- Children between the ages of 4 and 8 who have the capacity to understand and complete the clinical tests.
- No strabismus/squint seen on cover test and no known associated pathology.
- No more than 1D difference in refractive error (spherical component).
- At least 0.1 LogMAR vision in both eyes with or without refractive correction.
- No more than 1 LogMAR line difference in vision between both eyes, using either Crowded Kay Pictures or Crowded Keeler tests.

Eligible participants (anisometropic amblyopes and controls) were recruited by reviewing patient notes 1–2 weeks prior to their next follow-up appointment within the MYHT Orthoptic Department. The relevant participant information sheets (PIS) were then sent out to their postal addresses, allowing parents/guardians and potential participants to read and fully understand the purpose of the research. Signed consent was then obtained from parents/guardian and assent was obtained from participants at their follow-up appointment before testing. Participants with a refractive error wore their refractive correction during testing.

We used the post 1990 version of the FNS during testing. The starting distance with the FNS was 30 cm, which was measured from the participant’s eye level to the front of the FNS test box using a tape measure. The 3 test plates that comprised the FNS, were presented 3 times each at 30 cm to yield disparities of 600”, 300” and 150”, starting with the thickest plate (6 mm) first. At each level of disparity, participants were asked to choose which of the 4 squares on the plate had a circular shape ‘jumping’ out at them. Correct responses for 2 out of the 3 presentations were required to pass the level. This was to reduce the probability that participants were answering correctly by chance. If a participant could not pass the first plate, this was graded as nil stereopsis. When a participant achieved 150” disparity with the third plate at 30 cm, the plate was presented again 3 times with the plate being moved further back by 10 cm whenever the participant passed a particular distance, with the final distance being 80 cm as specified on the FNS. The threshold was recorded as the finest disparity at which 2 out of 3 presentations were identified correctly, or 20” if reached 80 cms. We were unable to physically control head movements of the participants due to limited time and equipment (e.g. head or chin rest), therefore we attempted to minimise motion parallax and a false number of positive results, through verbally reiterating to children throughout testing to keep their head as stationary as possible.

The test version for the TNO that was used was the TNO 15. Participants were presented with the TNO and red/green glasses were worn by participants to identify the patterns. The TNO was presented at 40 cm which was measured from the participant’s eye level to the open TNO test book, using a tape measure. Participants were tested using the ungraded plates first. If the participants failed to achieve correct responses on any of the ungraded plates then this was graded as nil stereopsis. The graded pages of the TNO were presented to the participants that passed the ungraded plates, and they were asked to identify which way the pattern was facing. If the participants were able to answer correctly on the ungraded plates but were unable to achieve correct responses on the graded plates, then this was graded as coarse stereopsis.

At each disparity level on the graded plates, 2 out of 2 correct responses were required to pass before moving onto the next disparity level to again rule out the probability of participants achieving correct responses by chance.

The stereoacuity values for both tests were converted to log arcseconds for the purpose of analysis to adapt the results into scale data and to retain information about the non-comparable scale between the TNO and FNS (Adams et al., 2009; Wallace et al., 2011; Van Doorn et al., 2014). Participants who demonstrated coarse/nil stereopsis had their stereoacuity graded as 0.3 log arcseconds above the lowest score achievable on each test (e.g. FNS = 3.08, TNO = 2.98). This method is commonly used in the analysis of stereoacuity data and the “nil” values were based on the 0.3 log arcsec intervals between disparity levels. One uniform figure for “nil” values could not have been used across both tests as the scale between the largest measurable disparity and nil differs between the FNS and TNO (Adams et al., 2009; Fawcett, Wang & Birch, 2005). Without this conversion, a direct comparison between the 2 tests would have been inaccurate.

A confidence interval of 5% was used. Statistical significance for all analyses was found if *p* < 0.05.

## Results

Forty-four participants were included in this study: 20 anisometropic amblyopes and 24 controls, with a mean age of 5.9 years. The anisometropic amblyopes were comprised of 7 females and 13 males, with a mean age of 6.6 years. The control group were comprised of 13 females and 11 males, with a mean age of 5.3 years.

Eleven anisometropic amblyopes were undergoing occlusion therapy or had received occlusion therapy previously. Tables 1 and 2 show the participant’s raw data.

**Table 1:** Raw data for anisometropic amblyopes.

Participant	Right visual acuity (LogMAR)	Left visual acuity (LogMAR)	Interocular Acuity Difference (LogMAR)	Glasses prescription right eye (dioptres)	Glasses prescription left eye (dioptres)

1	–0.100	0.250	0.350	+1.75/–0.75 × 180	+4.75/–1.00 × 175
2	0.450	0.100	0.350	+5.00/–0.75 × 82.5	+1.50
3	0.000	0.200	0.200	+2.00	+3.75
4	0.000	0.250	0.250	+5.50/–1.00 × 85	+8.50/–1.25 × 60
5	0.250	0.000	0.250	–3.00/+3.00 × 90	plano
6	0.100	–0.100	0.200	–3.50/–3.00 × 177	+0.75/–1.00 × 177
7	0.125	–0.100	0.225	+3.00/–0.50 × 90	+0.50/–0.50 × 90
8	0.200	0.000	0.200	+5.50/–0.50 × 20	+2.00/–0.25 × 170
9	0.000	0.200	0.200	+1.50	+4.50
10	0.100	–0.100	0.200	+2.25	+1.00
11	0.050	0.450	0.400	+1.00/–0.25 × 5	+4.50/–1.00 × 30
12	–0.100	0.100	0.200	+2.50/–1.00 × 10	+5.50/–4.50 × 5
13	0.075	0.500	0.425	+1.00	+2.50
14	0.050	0.425	0.375	+2.50	+4.50
15	–0.100	0.250	0.350	2.00/–0.25 × 123	+4.50/–0.25 × 59
16	0.175	–0.100	0.275	+3.00/–0.25 × 180	+0.75/–0.75 × 180
17	0.200	–0.075	0.275	+1.25/–2.00 × 180	∞/–0.50 × 180
18	0.025	0.550	0.525	+1.75	+6.00/–0.50 × 180
19	–0.100	0.350	0.450	+0.50	+7.25/–2.25 × 170
20	–0.100	0.300	0.400	+1.00	+5.50/–0.50 × 170

**Table 2:** Raw data for controls.

Participant	Right visual acuity (LogMAR)	Left visual acuity (LogMAR)	Difference between right & left visual acuity (LogMAR)	Glasses prescription right eye (dioptres)	Glasses prescription left eye (dioptres)

1	0.000	0.000	0.000	+5.25/–1.25 × 13	+5.25/–1.25 × 14
2	0.025	0.000	0.025	+3.25	+3.00
3	0.075	0.100	0.025	+7.00/–2.00 × 8	+7.25/–1.50 × 76
4	0.100	0.100	0.000	+5.00/–2.50 × 10	+4.75/–2.50 × 5
5	0.100	0.100	0.000	+4.50/–2.25 × 4	+4.50/–2.00 × 180
6	0.000	0.000	0.000	+1.75	+1.75
7	0.075	0.000	0.075	+2.50/–1.50 × 87.5	+1.50/–0.75 × 87.5
8	0.025	0.000	0.025	N/A	N/A
9	0.075	0.050	0.025	N/A	N/A
10	0.000	0.000	0.000	+7.00	+6.50
11	0.100	0.100	0.000	+2.00/–2.75 × 20	+2.00/–2.75 × 168
12	0.100	0.100	0.000	+5.00/–2.00 × 105	+4.25/–2.00 × 70
13	0.050	0.075	0.025	+7.75	+7.50
14	0.100	0.100	0.000	+2.75/–0.25 × 180	+3.75/–0.50 × 180
15	0.100	0.100	0.000	+6.00/–0.50 × 170	+6.00/–1.00 × 173
16	0.075	0.075	0.000	+2.25/–0.50 × 180	+2.25/–0.50 × 180
17	0.100	0.100	0.000	N/A	N/A
18	0.050	0.050	0.000	+2.50	+3.00
19	0.050	0.025	0.025	+0.50/–1.50 × 90	∞/–1.00 × 90
20	0.050	0.050	0.000	N/A	N/A
21	0.000	0.000	0.000	N/A	N/A
22	0.075	0.100	0.025	N/A	N/A
23	0.025	0.050	0.025	N/A	N/A
24	0.050	0.000	0.050	N/A	N/A

### 1) FNS vs. TNO

Table 3 shows that participants performed better on average with the FNS (2.17 ± 0.32) compared to the TNO (2.33 ± 0.40). Considering the standard error (SE) mean for both the FNS (0.05) and TNO (0.07), there is a 95% chance the wider population mean is within ±0.10 (2 × 0.05) for the FNS and ±0.14 (2 × 0.07) for the TNO RS.

**Table 3:** Mean stereoacuity showing the difference between FNS and TNO RS stereoacuity for all 44 participants.

	Group Statistics				

	Stereotest	N	Mean	Std. Deviation	Std. Error Mean

**Stereoacuity (Log arcsec)**	**FNS**	44	2.1677	0.31539	0.04755
	**TNO RS**	44	2.3323	0.43374	0.06539

N = number of participants.

Levene’s Test for Equality of Variances was used to assess whether the variance from the normal distribution between the two sample groups was equal. The conditions between the two tests vary significantly (0.018) in order to violate this assumption; therefore a non-parametric test was used. From this, we can take the p-value to be statistically significant (*p* = 0.045).

### 2) Controls vs. anisometropic amblyopes

**Figure 1:**
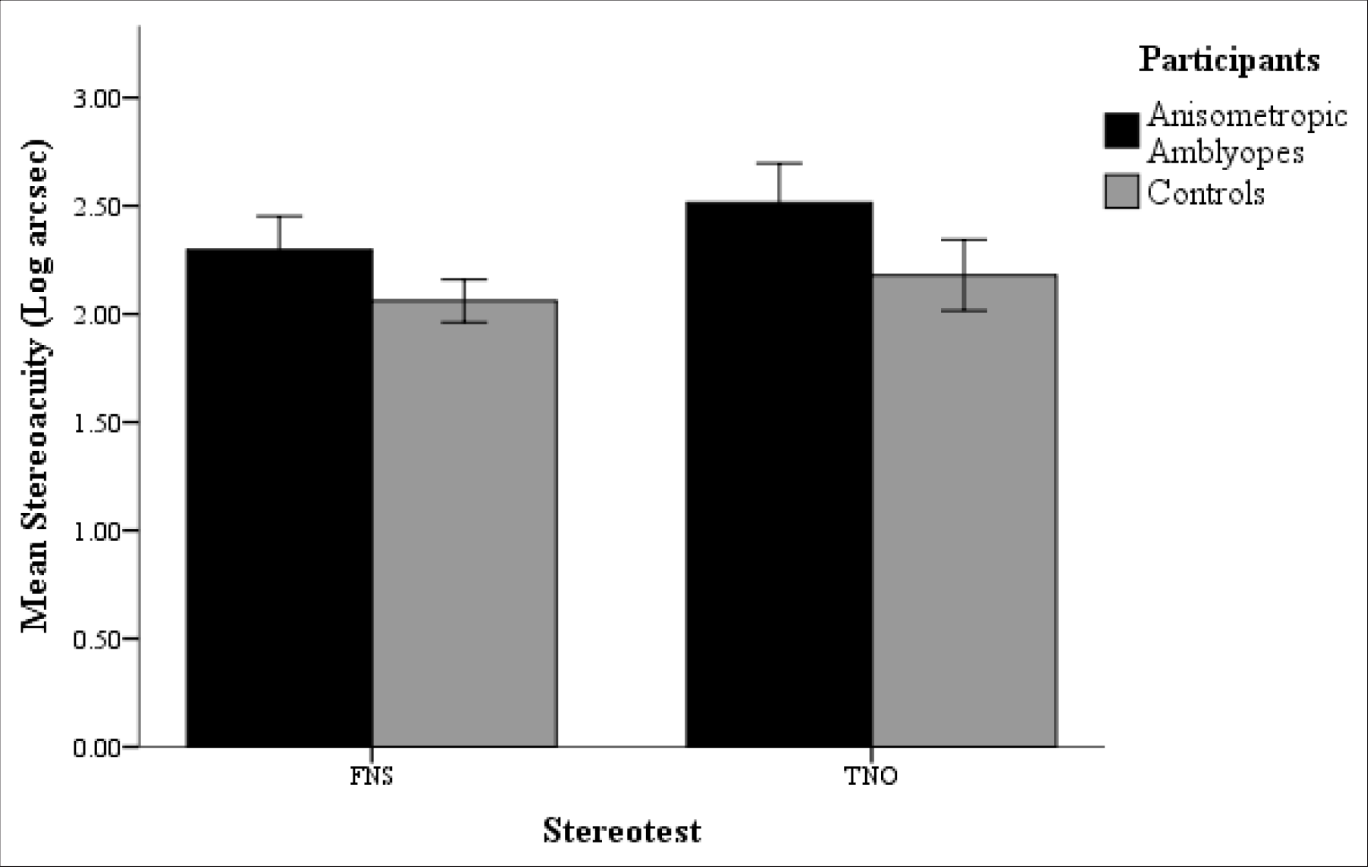
Difference in FNS and TNO stereoacuity between anisometropic amblyopes and controls, including SE bars (±2 SE).

As presented in Table 4 and Figure 1, it is evident that the mean stereoacuity achieved by the control group on the FNS (2.06 ± 0.24) and the TNO (2.18 ± 0.40), is better than the stereoacuity achieved by anisometropic amblyopes on the FNS (2.30 ± 0.35) and the TNO (2.52 ± 0.41), with a relatively high SD in all groups.

**Table 4:** Mean stereoacuity for the FNS and TNO RS between anisometropic amblyopes and controls.

	Group Statistics				

	Participants	N	Mean	Std. Deviation	Std. Error Mean

**FNS Stereoacuity (Log arcsec)**	**Anisometropic Amblyopes**	20	2.2960	0.34900	0.07804
	**Controls**	24	2.0608	0.24315	0.04963
**TNO RS Stereoacuity (Log arcsec)**	**Anisometropic Amblyopes**	20	2.5150	0.40688	0.09098
	**Controls**	24	2.1800	0.40217	0.08209

The SE mean for the controls (FNS = 0.05; TNO = 0.08), shows there is a 95% chance the wider population mean is within ±0.10 for the FNS and ±0.16 for the TNO RS. Whereas for the anisometropic amblyopes (FNS = 0.08; TNO = 0.09), there is a 95% chance the wider population mean is within ±0.16 for the FNS and ±0.18 for the TNO RS.

### 3) VA and stereoacuity

The relationship between VA in the amblyopic eye and TNO stereoacuity is fairly strong (correlation coefficient (*r*) = 0.544) with the value of *r* being closer to 1 than 0, showing a positive correlation (*p* = 0.013).

**Figure 2:**
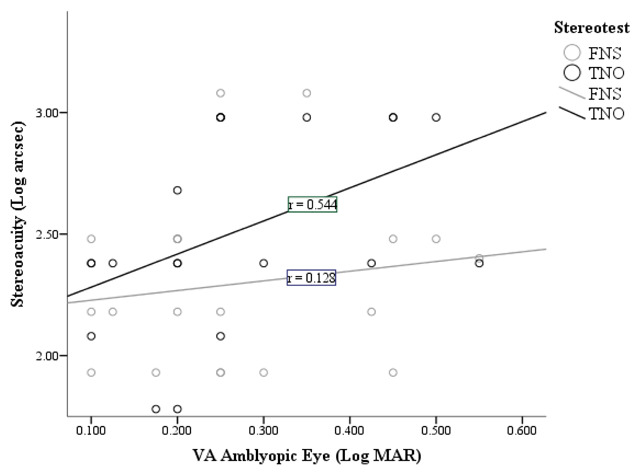
Correlation between VA in the amblyopic eye and stereoacuity in anisometropic amblyopes.

**Figure 3:**
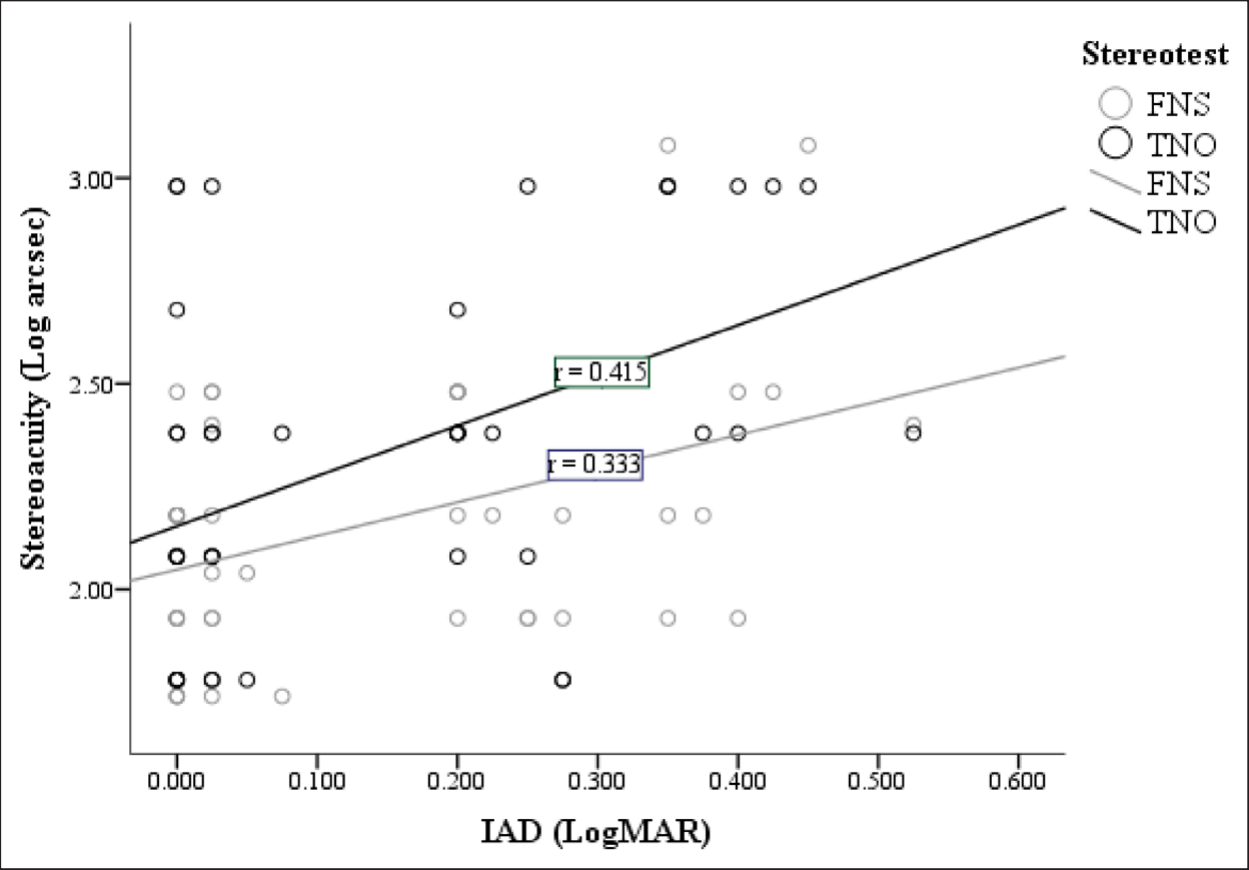
Correlation between IAD and stereoacuity for all 44 participants.

This is illustrated by the TNO best fit line (Figure 2), showing a positive correlation between increasing VA in the amblyopic eye and the threshold for TNO stereoacuity in anisometropic amblyopes. However, this is not the case regarding the relationship between VA in the amblyopic eye and FNS. There is no correlation between VA in the amblyopic eye and FNS stereoacuity in anisometropic amblyopes (*p* = 0.590) (Figure 3).

#### IAD and Stereoacuity in all participants

The majority of anisometropic amblyopes do not have a severe level of IAD, with 11 showing an IAD of 0.3 LogMAR or better. In relation to IAD and stereoacuity in all participants, it is noted that *r* = 0.333 for the FNS and *r* = 0.415 for the TNO. This shows there is a positive correlation between IAD and both stereotests when results from all 44 participants are analysed. It can also be seen in Figure 3 that the relationship is statistically significant between IAD and both the FNS (*p* = 0.027) and the TNO (*p* = 0.005).

### 4) Anisometropia and stereoacuity

There is no statistical significance between the degree of spherical anisometropia and FNS stereoacuity (*p* = 0.177).

**Figure 4:**
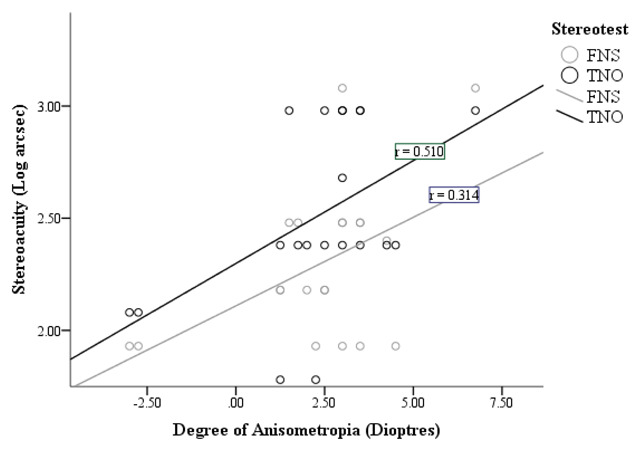
Correlation between the degree of spherical anisometropia (dioptre sphere) and stereoacuity in anisometropic amblyopes.

This is also indicated by the FNS best fit line shown in Figure 4. There is a positive correlation between increasing degree of spherical anisometropia and the threshold for FNS stereoacuity in anisometropic amblyopes; however it is not a statistically significant correlation.

From Figure 4, it can be seen that *r* = 0.510, demonstrating there is a strong correlation between the degree of spherical anisometropia and TNO stereoacuity (*p* = 0.022).

This is apparent from the TNO best fit line shown in Figure 4. As the degree of spherical anisometropia increases, the threshold for TNO stereoacuity also increases for anisometropic amblyopes.

## Discussion

The present study set out to assess the impact of anisometropic amblyopia on stereoacuity in children between the ages of 4 and 8; more specifically, whether the impact of anisometropic amblyopia is more detrimental when distinguishing 3D images using real depth stereotests or when distinguishing 3D images using random dot stereo tests. This included investigating the effect of VA level, IAD, degree of spherical anisometropia and the differences in stereoacuity between anisometropic amblyopes and their controls. Although statistical significant correlations were found, it can be seen that there was quite a lot of variability in the results for both tests.

### 1) FNS vs. TNO RS

One of the main findings of the present study was that all participants performed better with the FNS compared to the TNO. Statistically significant differences were detected between the FNS and TNO stereoacuities for all participants. This suggests that even in the absence of anisometropic amblyopia there is generally an increased difficulty associated with resolving patterns using the TNO. This outcome is similar to that discovered by Odell et al. (2009) who established significant stereoacuity degradation using Randot near and distance stereotests in comparison to the FNS and the Frisby-Davis 2 (FD2) tests. Thus, concluding that the FNS and the TNO measure two separate aspects of stereopsis. However, they conducted their study using 182 participants with a range of strabismic conditions, making it difficult to decipher whether the stereoacuity differences were due to the stereotests used or the underlying strabismus. In the present study, it could be argued that the statistical significance found is relatively low and may not be significant when it comes to clinical practice. Although the standard deviation (SD) is fairly high for both groups, this could be due to the small sample size.

It is well known that the TNO has a higher threshold in comparison to the FNS due to the design. The TNO uses red/green glasses worn by patients to distinguish different shapes ranging in difficulty. The red/green glasses give rise to dissociation and rivalry between the two eyes as each eye is looking through a different colour (Read, 2015). This design in itself makes it more difficult for the eyes to work together regardless of any underlying ocular condition that may be present, such as anisometropic amblyopia. This could account for the controls obtaining reduced stereoacuity using the TNO also. Although the FNS does not use other aids that may dissociate the two eyes, motion parallax may have come into play if the children moved their head during testing which allows for monocular cues. It has been suggested that such tests as the FNS that can give rise to motion parallax, should be tested monocularly and if the same result is achieved, the binocular score is an inaccurate finding of stereoacuity (Read, 2015; Costa et al., 2010).

The results found in the present study generally show participants to achieve higher scores with the FNS. The FNS was not tested monocularly as a comparison so it is a possibility that motion parallax could have contributed to the stereoacuity scores, regardless of continued encouragement in order for participants to keep stationary.

### 2) Controls vs. anisometropic amblyopes

Another finding of the present study is the difference between the stereoacuity results achieved by the anisometropic amblyopes and the controls on both the FNS and TNO. Although all participants performed poorly on the TNO compared to the FNS, it is evident that the controls performed significantly better on both tests when compared to the anisometropic amblyopes. Differences between both groups on the FNS and the TNO were shown to be of statistical significance. The results of the present research adds to previous research demonstrating that monocular blur has a statistically significant detrimental effect on stereoacuity (Odell et al., 2009; Singh et al., 2015; Goodwin & Romano, 1985; Chen et al., 2013; Ying et al., 2013; Brooks, Johnson & Fischer, 1996).

Aside from the present study, there are a limited amount of studies found that investigate the effect of true anisometropic amblyopia on stereoacuity using actual, paediatric anisometropic amblyopes and their controls for comparison. A number of these studies used only one stereotest during their research, whereas this study compared the response of two different stereotests. There are many similar studies that have tested the effect of an ocular deficit (e.g. strabismic and anisometropic amblyopia, stimulus deprivation amblyopia, induced anisometropic amblyopia, occlusion therapy) on adults as well as children, producing similar results (McKee, Levi & Movshon, 2003; Brooks, Johnson & Fischer, 1996; Rutstein & Corliss, 1999).

### 3) VA and stereoacuity

VA and IAD are directly correlated to the level of achievable stereoacuity in participants, based on the improvement of stereoacuity after VA improves with refractive correction, with or without occlusion therapy (Stewart et al., 2013; Lee, Seo & Baek, 2013; Mitchell, Howell & Keith, 1983; Harvey et al., 2008). Participants in the present study were tested at one point in time. As a result, there was no way to determine whether stereoacuity thresholds achieved by participants undergoing occlusion therapy had improved since their previous clinic visit. It is clear there is a correlation between the level of VA in the amblyopic eye and TNO stereoacuity. Thus, further demonstrating that better VA in the amblyopic eye results in improved stereoacuity thresholds. This was also the case between IAD and TNO stereoacuity, in addition to IAD and FNS stereoacuity from the data taken from all 44 participants. This proves that stereoacuity thresholds increase as IAD increases.

Although stereoacuity with the FNS appeared to be reduced in the presence of poor amblyopic VA and high IAD, no statistical significance was found between amblyopic VA and FNS stereoacuity, and also between IAD and FNS stereoacuity in anisometropic amblyopes. This could be due to the FNS measuring the ability to perceive coarse texture. Therefore it does not require anisometropic amblyopes to utilise the first-order processing of fine detail; it would mainly require the second-order processing of the more coarse information to elicit a good result. As a consequence, anisometropic amblyopes may have the ability to achieve high stereoacuity thresholds with the FNS and struggle to achieve similar stereoacuity thresholds with the TNO (McColl, Ziegler & Hess, 2000; McColl & Mitchell 1998; Giaschi et al., 2013). Conversely, there was also no statistical significance found between IAD and TNO stereoacuity in anisometropic amblyopes. This finding is unusual and opposes previous research that has shown statistical significance between IAD and stereoacuity thresholds (Mitchell, Howell & Keith, 1983; Harvey et al., 2008; Schmid & Largo, 1986). Previous studies have shown that anisometropic amblyopes find it more challenging to identify the finer texture of a pattern, as this requires first-order processing which may be the weaker processing system for anisometropic amblyopes (McColl, Ziegler & Hess, 2000; McColl & Mitchell 1998; Giaschi et al., 2013). Eleven of the 20 anisometropic amblyopes in this study had an IAD of 0.3 LogMAR or better, which is not as severe as the IAD established in the aforementioned studies. This may have some bearing on the lack of statistical significance between IAD and TNO stereoacuity observed in the anisometropic amblyopes within the present study.

### 4) Anisometropia and stereoacuity

The degree of anisometropia in relation to stereoacuity was analysed solely on the spherical element (hypermetropia and myopia) of anisometropia (Townshend, Holmes & Evans, 1993). The astigmatic component of participant’s glasses prescriptions were left out as evidence shows astigmatic anisometropia does not have a detrimental effect on stereoacuity (Dobson et al., 2008; Dadeya, Kamlesh & Shibal, 2001).

FNS stereoacuity thresholds degrade as the degree of spherical anisometropia increases, with no statistical significance noted. This is likely due to a random variation within the results. Previous research reports a strong relationship between the degree of anisometropia and stereoacuity, where as little as +1D of spherical anisometropia was enough to degrade stereoacuity (Ying et al., 2013; Weakley, 2001; Levi, McKee & Movshon, 2011; Rutstein & Corliss, 1999). However, many of the tests used were not real depth tests like the FNS which appears more resilient to the effects of anisometropia (Leske, Birch & Holmes, 2006).

The correlation between degree of spherical anisometropia and TNO, appear to support the previous studies that found a strong correlation between degree of anisometropia and stereoacuity (Ying et al., 2013; Weakley, 2001; Levi, McKee & Movshon, 2011; Rutstein & Corliss, 1999; Singh et al., 2015).

### Limitations of the study

One of the main limitations of the study was the small sample size. Fifty-two participants were recruited, however 8 participants were excluded due to underlying pathlogy or microstrabismus. This may not have allowed for enough power to find significant correlations. Due to this sample size, the results obtained from each test group may not accurately relate to the wider paediatric population.

Data was collected by 3 different experimenters. Each experimenter was given an instruction sheet outlining the steps to take when collecting the data to try and assure all participants were tested in a standardised way. However, it is possible small differences in the way each experimenter explained test instructions to participants may have affected the results achieved.

An additional weakness to the study was the inability to control head movements of participants during testing with the FNS, attributable to lack of necessary equipment. Although experimenters tried to counteract for this, motion parallax could account for some participants achieving higher stereoacuity results on the FNS.

## Conclusion

Anisometropic amblyopia can have a significant detrimental effect on stereoacuity in children. This impact on stereoacuity appears to be more apparent the more the VA is reduced, when IAD is large and when the degree of spherical anisometropia is greater. The reduction in stereoacuity found in the presence of these factors is more pronounced when trying to resolve the fine texture of 2 horizontally displaced images using the TNO, compared to identifying a coarse, single 3D image from the thickness of a plate, producing the disparity and the horizontal component when using the FNS. The controls within this study also performed worse on the TNO in comparison to the FNS. Thus demonstrating real depth stereotests such as the FNS, can be used to detect the absence or presence of stereopsis in anisometropic amblyopia, even in more severe cases; whereas random dot stereotests such as the TNO, can detect very subtle binocular abnormalities. However, clinicians need be aware that due to this, the results of each stereotest are not interchangeable within the clinical setting as it could provide an inaccurate clinical picture when assessing how treatment (e.g. occlusion therapy) is progressing in terms of visual function.
